# Antimicrobial Efficacy of Aqueous Ozone and Ozone–Lactic Acid Blend on *Salmonella*-Contaminated Chicken Drumsticks Using Multiple Sequential Soaking and Spraying Approaches

**DOI:** 10.3389/fmicb.2020.593911

**Published:** 2020-12-14

**Authors:** Ameer Megahed, Brian Aldridge, James Lowe

**Affiliations:** ^1^Department of Veterinary Clinical Medicine, College of Veterinary Medicine, The University of Illinois at Urbana-Champaign, Urbana-Champaign, IL, United States; ^2^Department of Animal Medicine, Internal Medicine, Faculty of Veterinary Medicine, Benha University, Benha, Egypt

**Keywords:** aqueous ozone, ozone–lactic acid blend, sequential soaking, sequential spray, *Salmonella*, bacterial load, chicken parts

## Abstract

Ozone (O_3_) is an attractive alternative antimicrobial in the poultry processing industry. The optimal operational conditions of O_3_ for improving food safety concerns are poorly understood. The main objective of this study was therefore to characterize the microbial killing capacity of aqueous O_3_ and O_3_–lactic acid blend (O_3_–LA) at different operational conditions on chicken drumsticks contaminated with high *Salmonella* load using sequential soaking and spraying approaches. Four hundred forty-eight chicken drumsticks (280–310 g) were soaked into two-strain *Salmonella* cocktail, and the initial load on the surface of the skin was 6.9-log_10_ cell forming unit (CFU)/cm^2^ [95% confidence interval (CI), 6.8–7.0]. The contaminated drumsticks were then sequentially (10×) soaked and sprayed with aqueous O_3_ (8 ppm) and O_3_–LA. Following O_3_ exposure, quantitative bacterial cultures were performed on the post-soaking and post-spraying water, skin surface, and subcutaneous (SC) of each drumstick using 3M^TM^ Petrifilm^TM^ Rapid Aerobic Count Plate (RAC) and plate reader. The average killing capacity of aqueous O_3_/cycle on the skin surface was 1.6-log_10_/cm^2^ (95% CI, 1.5–1.8-log_10_/cm^2^) and 1.2-log_10_/cm^2^ (95% CI, 1.0–1.4-log_10_/cm^2^), and it was 1.1-log_10_/cm^2^ (95% CI, 0.9–1.3-log_10_/cm^2^) and 0.9-log_10_/cm^2^ (95% CI, 0.7–1.1-log_10_/cm^2^) in SC for soaking and spraying approaches, respectively. Six sequential soaking and seven sequential spraying cycles with ozonated water of 8 ppm reduced the heavy *Salmonella* load below the detectable limit on the skin surface and SC of drumsticks, respectively. Addition of LA seems to increase the microbial killing capacity of aqueous O_3_ with average differences of 0.3-log_10_/cm^2^ (*P* = 0.08) and 0.2-log_10_/cm^2^ (*P* = 0.12) on the skin surface using soaking and spraying approaches, respectively. Aqueous O_3_ did not cause any significant changes in the drumstick skin color. The *Salmonella* load of < 4.5-log_10_/cm^2^ was a strong predictor for the reduction rate (*P* < 0.001, *R*^2^ = 0.64). These results provide important information that helps the poultry processing facilities for selecting the optimal operational strategy of O_3_ as an effective antimicrobial.

## Introduction

Chicken meat is the second most popular meat in the world, which accounts for ∼30% of meat production preceded by pork with 38% of the total meat production. In the United States, poultry meat is the most popular consumed meat, and the consumption has steadily increased in recent years. The annual poultry meat consumption per capita has increased from 27 lb in 1970 to 60 lb in 2005 (National Chicken Council, 2011). The reasons for the popularity of this kind of meat are the competitive price, absence of cultural and religious obstacles, fast preparation, low fat content, and the high nutritional value (Food and Agriculture Organization of the United Nations [FAO], 2014). The chicken meat market has changed from whole birds to chicken parts over the years. In the last decades, 83% of broilers were marketed as whole birds, whereas 15% were marketed as parts. In 2009, 12% of broilers were consumed as whole birds, and 42% as parts (National Chicken Council, 2011). Chicken meat supports bacterial growth due to high water content, large amounts of variable nutrients, and high pH resulting in short shelf-life of products (Lawrie, 1998). In the last few years, consumers are more concerned about food safety than ever before. The US Department of Agriculture, Food Safety and Inspection Service (USDA-FSIS) recommendations are to control pathogens prevalence in the products that are most often purchased by consumers (United States Department of Agriculture, Food Safety, and Inspection Service, 2015). The new standards include culture for foodborne pathogens at the end of the chilling line and in the cut-up room (United States Department of Agriculture, Food Safety, and Inspection Service, 2015).

In the United States, foodborne diseases cause 8 million cases annually, 128,000 hospitalizations, and 3,000 deaths (Centers for Disease Control and Prevention, 2011). The total annual economic burden of illness calculated based on the enhanced cost-of-illness model is $77.7 billion (Scharff, 2012). *Salmonella* is the most frequently reported cause of foodborne illness in the United States with roughly 1.2 million cases annually, ∼20,000 hospitalizations, and ∼400 deaths (Scallan et al., 2011). The total annual cost including medical costs and loss of productivity has been estimated to be about 3.3 billion (Hoffmann et al., 2012). *Salmonella* is the most commonly associated pathogen with poultry meat and poultry products (Hoffmann et al., 2012). Consequently, it is considered a good indicator of the hygienic conditions in the poultry processing facilities (Kottwitz et al., 2010; Silva et al., 2010; Jakobsen et al., 2012). The chicken carcass is considered spoiled or deteriorated when the microbial count reaches 10^6^–10^9^ cell forming unit (CFU)/cm^2^ (Russell, 2001). Therefore, the safe elimination or, at least, reduction to a safe level is an immediate challenge in the poultry processing facilities. Currently, bacterial contamination is removed or controlled during the carcass processing using a combination of thermal treatment, water, and chemical antimicrobials, such as peracetic acid (PAA) (Northcutt et al., 2005; Dittoe et al., 2019). However, these chemical antimicrobials are faced with challenges including last for several hours, and most of them can be toxic before and after the breakdown (Megahed et al., 2018a,b, 2019). Additionally, these chemical antimicrobials can produce undesired color and texture effects and development of off-flavors (Qiao et al., 2002). Accordingly, the utilization of other antimicrobials with a high killing capacity, short half-life, and decompose to non-toxic molecules is a priority goal for the poultry industry.

Ozone (O_3_) is known for its strong oxidative power with an oxidative potential of 2.07 V, nearly twice the oxidizing potential of chlorine (1.36) and greater than the oxidizing potential of PAA (1.81) (Russel et al., 1999). Because of its strong oxidation potential, O_3_ is very toxic to bacteria, even at concentrations as low as 0.01 ppm (Qingshi et al., 1989). Additionally, O_3_ did not show any negative impact on the final appearance of poultry products (Mancini and Hunt, 2005). In 2002, the USDA approved O_3_ as a safe and suitable ingredient used in the production of meat and poultry (United States Department of Agriculture, 2002). In 1979, Yang and Chen reported approximately 3-log_10_ microbial reduction on chicken necks immersed in ozonated water of 2.48 ppm for 9 min exposure time (Yang and Chen, 1979). In another study, ozonated water did not show significant microbial reduction rate (1-log_10_) on chicken carcasses chilled with water containing 3.0–4.5 ppm of O_3_ for 45 min exposure time (Sheldon and Brown, 1986). This indicated that the microbial killing capacity of aqueous O_3_ on *Salmonella*-contaminated chicken carcasses is still an unresolved issue. Among the potential decontamination protocol is the blend of organic acid solutions and oxidizing agents that have been evaluated on the poultry products and showed variable results. A combination of lactic acid (LA) and PAA reduced *Escherichia coli* O157 and organic materials to a safe level in the fresh-cut leafy vegetables (Haute et al., 2015). To the best of our knowledge, no study evaluated the combination of O_3_–LA for reducing the pathogens prevalence and levels in the chicken cuts. Accordingly, the primary objective of the present study was to characterize the microbial killing capacity of aqueous O_3_ and O_3_–LA blend at different operational conditions on chicken drumsticks contaminated with high *Salmonella* load using sequential soaking and spraying approaches. We also hypothesized that the bacterial load has a significant impact on the microbial killing capacity of aqueous O_3_ (Megahed et al., 2018a,b). The secondary objective was therefore to use the segmented linear regression to determine the effect of skin surface microbial load on the microbial killing capacity of aqueous O_3_.

## Materials and Methods

### Preparation of Inoculated Samples

Four hundred forty-eight chicken drumsticks with an average weight ranging from 280 to 310 g obtained from local grocery stores were used in this study. Sample size was determined from the effect size and variation observed in a preliminary, unreported trial. Sampling at each intervention was based on a statistical power of 80%, an expected standard deviation of 0.8-log_10_ CFU/ml, and a desired reduction rate of 0.7-log_10_ CFU/ml. All drumsticks were kept in a freezer (−20°C) from the time of reception until used. Before the inoculation process, the enclosed drumsticks bags were thawed in a refrigerator at 4°C for 12 h and then held at room temperature for 1 h.

To confirm no *Salmonella* background on the drumsticks before inoculation, each drumstick sample was first rinsed with buffered peptone water (BPW; Difco, BD, Sparks, MD, United States) in a labeled sterile Nasco WHIRL-PAK bag by manually shaking the bag for 1 min. An area of approximately 6 cm^2^ of the skin surface and subcutaneous (SC) was also swabbed with sterile cotton swabs (Pur-Wraps^®^; Puritan Medical Products, Guilford, ME, United States). One ml of the rinsate solution and swabs were added to 9 ml of BPW. One ml of the mixture was streaked on Tryptic Soy Agar plates (TSA W/5% sheep blood agar; Remel, Lenexa, KS, United States) for bacterial identification using matrix-assisted laser desorption ionization-time of flight (MALDI-TOF) mass spectrometry (Bruker Daltonik, Bremen, Germany) at the Veterinary Diagnostic Laboratory of the University of Illinois Urbana-Champaign. Detailed bacterial identification using MALDI-TOF mass spectrometry has been previously mentioned in our previous work (Megahed et al., 2018a,b). Drumsticks with background bacteria were thoroughly washed with aqueous O_3_ (8 ppm) in a labeled sterile Nasco WHIRL-PAK bag until we get bacteria-free drumsticks. The clean drumsticks were then held in a laminar airflow hood (Baker, Sanford, ME, United States) for 30 min to ensure O_3_ destruction (Hirahara et al., 2019).

Avirulent live *Salmonella typhimurium* and *Salmonella choleraesuis* (aSTC) vaccine (Enterisol^®^ *Salmonella* T/C vaccine; Boehringer Ingelheim Vetmedica, St. Joseph, MO, United States) was used as a source of two *Salmonella* strains. The two *Salmonella* strains were revitalized and activated to be used as a model to characterize the *Salmonella* killing capacity of aqueous O_3_ (Wigley et al., 2005; Lottenbach et al., 2013). *Salmonella* was recovered using BPW in 50 ml sterile conical polypropylene tubes equipped with a lid (Thermo Fisher Scientific, Waltham, MA, United States) at 1:9 (vaccine/BPW) ratio. The mixture was incubated at 37°C for 48 h to ensure that all injured cells were recovered. A serial 10-fold dilution was used in order to determine the level of inoculum by spreading 1 ml from each dilution on 3M^TM^ Petrifilm^TM^ Rapid Aerobic Count Plate (RAC; 3M^TM^ Microbiology, St. Paul, MN, United States) using 3M^TM^ Petrifilm^TM^ spreader (3M^TM^ Microbiology, St. Paul, MN, United States). 3M^TM^ Petrifilm^TM^ RAC was used for enumeration of aSTC as it is accurate, easy to use, more efficient, commercially available, enumeration medium has a quick turn-around time, and reading results are automated (Nelson et al., 2013; Kumar et al., 2020). One ml of the mixture was also spread on TSA for bacterial identification to confirm the presence of only aSTC using MALDI-TOF mass spectrometry. A *Salmonella* cocktail mixture of an inoculum level of 10^7^ CFU/ml was used for contamination of drumsticks.

Clean drumsticks were soaked in aSTC cocktail with an inoculum level of 0^7^ CFU/ml for 5 min, the worst-case scenario, at a room temperature of 20°C and relative humidity of 55–60%. The soaked samples were aerated in a clean laminar airflow hood (Baker, Sanford, ME, United States) for 30 min to evaporate the peptone water and allow for bacterial attachment. After drying, aseptic techniques were used to place each drumstick into a labeled sterile Nasco WHIRL-PAK bag. The initial inoculum on the surface of the skin was then detected by swabbing the skin surface with sterile cotton swabs. The culture protocol and cell count quantification were similar as that described above.

### O_3_ Generation

The aqueous O_3_ with a concentration of 8 ppm was obtained using an OOG1 × 0 O_3_ generator manufactured by Origin, Inc. (Princeton, NJ, United States) as previously described in our work (Megahed et al., 2018a,b, 2019).

### Sequential Soaking Approach

This experiment was designed to characterize the microbial killing capacity of aqueous O_3_ and O_3_–LA on aSTC heavily contaminated drumsticks using multi-sequential soaking approach. Fifty inoculated drumsticks were randomly assigned into four groups, aqueous O_3_ treatment (*n* = 15), O_3_–LA treatment (*n* = 15), positive control (*n* = 15), and negative control (*n* = 5). The negative control drumsticks were used to detect the presence of background *Salmonella.* The aqueous O_3_-treated drumsticks were sequentially soaked (10-serial washes) with 500 ml of water containing 8 ppm of O_3_ for 4 min exposure each (Megahed et al., 2018a,b, 2019). A waiting time of 30 min between cycles was performed in order to ensure the destruction of O_3_ (Hirahara et al., 2019). The O_3_–LA-treated drumsticks were sequentially soaked (10-serial washes) with 500 ml of ozonated water containing 0.3% L-lactic acid (Sigma Aldrich, St. Louis, MO, United States) for 4 min exposure. The concentration of LA was selected to achieve a pH range from 2 to 3 to provide an optimum condition to maximize the oxidative power of O_3_ (Van Netten et al., 1994). A volume of 500 ml was used as the optimal volume enough to completely cover the drumstick in the WHIRL-PAK bag. The temperature of the ozonated water was 10–12°C. The bags were gently shaken for the exposure period. The positive control drumsticks were sequentially soaked (10-serial washes) with 500 ml autoclave sterilized distilled water (DW) for the same exposure time.

Two milliliters of soaking water was aspirated each soaking cycle and used for culture. One milliliter was spread directly on RAC Petrifilm^TM^ plate, and 1 ml was then serially diluted (fivefold dilutions) in 9 ml of BPW. An area of approximately 6 cm^2^ of the skin surface and SC was also swabbed with sterile cotton swabs at different points each soaking cycle. Each swab was washed in 10 ml of BPW, and 1 ml was then serially diluted (threefold dilutions) in 9 ml of BPW. One milliliter from each dilution was spread on RAC Petrifilm^TM^ plate. All RAC Petrifilm^TM^ plates were incubated at 37°C for 24 h. One milliliter from each dilution was also grown on TSA for bacterial identification using MALDI-TOF mass spectrometry for confirming only *Salmonella* isolates. The colony forming units were counted using an automated counter (3M Petrifilm Plate Reader; 3M^TM^ Microbiology, St. Paul, MN, United States). The plates were incubated for further 2–3 days in order to remove the effect of recovery of injured cells. To accommodate the effective reading range of the plate reader (maximum reading < 999/plate), only plates with 30–300 colonies were used for calculating the bacterial reduction factor (RF).

### Skin Color

The color of the skin surface of aqueous O_3_ and O_3_–LA-treated drumsticks was measured quantitatively by determining the RGB measurement using the ImageJ software program (version 1.50i; National Institutes of Health, Bethesda, MD, United States). Digital images were taken for the drumsticks each washing cycle using the Canon PowerShot SX420 IS 20.0 MP camera (Canon USA Inc., Lake Success, NY, United States). The drumsticks were hanged vertically in front of a white background and exposed to diffuse light to ensure the same amount of light distributed over the drumstick. A region of interest (ROI) of 20 × 20 mm was defined.

### Determination of Ozonated Water Volume for Spraying Approach

This experiment was designed to determine the reliable ozonated water volume used for spraying approach to decontaminate the aSTC-contaminated drumsticks. The inoculated-drumsticks were sprayed with 100 (*n* = 6), 200 (*n* = 6), 300 (*n* = 6), and 500 ml (*n* = 6) of water containing 8 ppm of O_3_ using a veterinary spray nozzle (Sunny Farms, San Francisco, CA, United States) attached to 500-cc syringe. The spray protocol was done by one operator (AM) in order to eliminate the potential for subjective operator-to-operator differences. The drumsticks were hanged in labeled sterile Nasco WHIRL-PAK bags and sprayed for 5 s. The spray solution was equally distributed over the surface of drumsticks by locating the syringe tips 5 cm above the drumsticks. The control drumsticks were sprayed with 100 (*n* = 6), 200 (*n* = 6), 300 (*n* = 6), and 500 ml (*n* = 6) DW. The culture protocol from spraying water, skin, and SC swabbing and quantification of cell count were similar as that described above in the first experiment.

### Sequential Spraying Approach

This experiment was designed to characterize the microbial killing capacity of aqueous O_3_ and O_3_–LA blend on the aSTC-contaminated drumsticks using multi-sequential spraying approach. The control and treated drumsticks were sequentially sprayed (10-serial sprays) with 100 ml. The spraying protocol was similar as that described above. The culture and cell count quantification were similar as that described above.

### Effect of Bacterial Load

This experiment was designed to characterize the impact of bacterial load on the microbial killing capacity of aqueous O_3_ using soaking and spraying approaches. Three hundred drumsticks were used for both washing (150) and spraying (150) approaches. The skin surfaces of drumsticks were contaminated with five different aSTC loads (3.0, 4.0, 5.0, 6.0, and 7.0-log_10_/ml). The soaking, spraying, culture, and cell count quantification protocol were similar as that described above.

### Data and Statistical Analyses

The log_10_ aSTC density for each drumstick was calculated using the formula presented in the ASTM method E2871-12 (ASTM E2871–12, 2012), as follows:

$$l\,o\,g_{10}\,\left(\frac{c\,f\,u}{m\,L}\right)=l\,o\,g_{10}\,\left\{\left(\frac{c\,f\,u}{v\,o\,l\,u\,m\,e\,p\,l\,a\,t\,e\,d}\right)\times \left(\frac{w\,a\,s\,h\,i\,n\,g\,s\,o\,l\,u\,t\,i\,o\,n\,v\,o\,l\,u\,m\,e}{d\,i\,l\,u\,t\,i\,o\,n}\right)\right\}$$

We added 1 in such way log_1__0_(x + 1) in order to overcome the problem of log-transformation of 0 CFUs. RF was also calculated for the first washing and/or spraying cycle by using an equation presented in the ASTM method E2871-12 (ASTM E2871–12, 2012), as follows:

$$l\,o\,g_{10}\,R\,F=l\,o\,g_{10}\,c\,o\,n\,t\,r\,o\,l-l\,o\,g_{10}\,t\,r\,e\,a\,t\,e\,d$$

For subsequent soaking and spraying cycles, RF was calculated by subtraction of the aSTC count of the current cycle from the aSTC count of the previous cycle.

Statistical analyses were performed using SAS 9.4 software (SAS Inc., Cary NC, United States), RStudio (version 1.1.383; R Studio, Inc., Boston, MA, United States), and Excel spreadsheet (Microsoft Corporation, Redmond, WA, United States). Data were expressed as median and range or as mean ± 95% confidence interval (CI) based on testing for normality by calculating the Shapiro–Wilk Statistic or based on testing equality of variances using Levene’s test. *P* < 0.05 was considered significant. For comparison between groups, Kruskal–Wallis One Way Analysis of Variance on Ranks was used for non-normal distribution or unequal variances data. For *post hoc* comparisons, *P*-values were adjusted for multiple comparisons according to Tukey. Mixed models analysis (PROC MIXED) of variance was used to detect differences in log_10_ RF between treatments (three levels; O_3_, O_3_–LA, and DW), sequential cycles, and the interaction between treatment and cycles. Cycles were included in the model as a repeated variable. Drumstick nested within treatment was included in the model as a random effect whenever the between subjects variation was determined to be significant. Whenever the F-test was significant, Tukey-adjusted *P*-values were used to assess differences between different treatments at a specific soaking or spraying cycle and between sequential cycles.

Univariate regression was used to characterize the association between RF and ozonated water volume for spraying approach. Mixed model segmented linear regression (PROC NLMIXED, SAS 9.4) was used to characterize the relationship between the bacterial reduction rate and the aSTC load (Gonçalves et al., 2016; Trefz et al., 2018). A mixed model approach using the adaptive Gauss–Hermitage quadrature approximation method for the marginal likelihood function was applied using quasi-Newton optimization, with μ0 representing the random background coefficient assuming the distribution of random effects to be normal with mean (μ0) = 0 and variance = s2e. The model equations assumed a constant value for RF when aSTC load < the cut point identified by segmented regression (Xc) and a negative linear relationship between the bacterial reduction rate and the aSTC load when aSTC load < Xc, such that: RF = (b0 + μ0) when aSTC load ≥ Xc and [b0 + μ0 − b1 × (aSTC load-Xc)] when aSTC load ≤ Xc. The segmented linear regression approach permitted the objective identification of increasing aSTC reduction when aSTC load started to decrease.

## Results

The background bacteria identified by direct MALDI-TOF mass spectrometry were *Bacillus thermosphacta*, Enterobacteriaceae, and *E. coli*. The results of direct MALDI-TOF mass spectrometry confirmed the presence of only aSTC during the whole experiment. The average of the initial bacterial load after contamination with aSTC was 6.94-log_10_ (95% CI, 6.87–7.0).

### *Salmonella* Reduction by Sequential Soaking Approach

**FIGURE 1:**
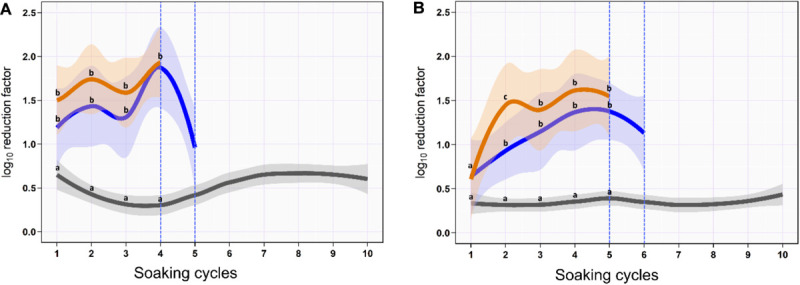
Mean and 95% CI of the log_10_ reduction in *Salmonella typhimurium*–*choleraesuis* (aSTC) cell count on the skin surface **(A)** and subcutaneous **(B)** of drumstick soaked with distilled water (gray), ozonated water of 8 ppm (blue), and ozone–lactic acid blend (orange) using multi-sequential soaking approach. The vertical dashed blue line indicates that no aSTC was detected.

On the skin surface, the first soaking cycle using ozonated water of 8 ppm reduced the aSTC load from 6.9 to 5.7-log_10_/cm^2^ with an average RF of 1.2-log_10_/cm^2^ (mean, 95% CI, 0.9–1.5). The average RF was then gradually increased in the subsequent soaking cycles until it reached a peak of 2.5-log_10_/cm^2^ (95% CI, 2.1–2.8) at the fourth soaking cycle, where the aSTC load reduced from 2.8 to 0.4-log_10_/cm^2^. Five sequential soaking cycles were sufficient to reduce the heavy bioload of aSTC (6.9-log_10_/ml) below the detectable limit on the skin surface (Supplementary Table S1 and Figure 1A). However, the O_3_–LA blend reduced the aSTC load from 6.9-log_1__0_ to 5.4-log_10_/cm^2^ in the first soaking cycle with an average RF of 1.5-log_10_/cm^2^ (95% CI, 1.1–1.9), reaching a peak of 1.9-log_10_ (95% CI, 1.5–2.4) at the fourth soaking cycle, where the aSTC load decreased from 1.9-log_10_/cm^2^ to the level below the detectable limit. O_3_–LA blend showed higher aSTC killing capacity than aqueous O_3_ with a total average difference of 0.3-log_10_/cm^2^ (*P* = 0.08), where four sequential soaking cycles were sufficient to reduce the heavy bioload of aSTC below the detectable limit (Supplementary Table S1 and Figure 1A).

In SC, ozonated water of 8 ppm reduced the aSTC load 0.6-log_10_ (95% CI, 0.3–1.0) in the first soaking cycle, where the aSTC load decreased from 6.8 to 6.2-log_10_. The average RF was then gradually increased in the subsequent soaking cycles until it reached a peak of 1.4-log_10_ (95% CI, 0.8–2.0) at the fifth soaking cycle, where the aSTC load was decreased from 3.9-log_1__0_ to 2.5-log_10_. Six sequential soaking cycles were sufficient to reduce the heavy bioload of aSTC (6.8-log_10_) below the detectable limit (Supplementary Table S1 and Figure 1B). However, O_3_–LA blend reduced the aSTC load from 6.8 to 6.2-log_10_ in the first soaking cycle with an average RF of 0.6-log_10_ (95% CI, 0.2–1.0), reaching a peak of 1.6-log_10_ (95% CI, 1.0–2.2) at the fifth soaking cycle, where the aSTC load was decreased from 2.5-log_1__0_ to 0.9-log_10_. Addition of LA with low concentration to aqueous O_3_ seems to intensify the O_3_ microbial killing capacity, where five sequential soaking cycles were sufficient to reduce the heavy bioload of aSTC below the detectable limit (Supplementary Table S1 and Figure 1B).

### Surface Damage

**FIGURE 2:**
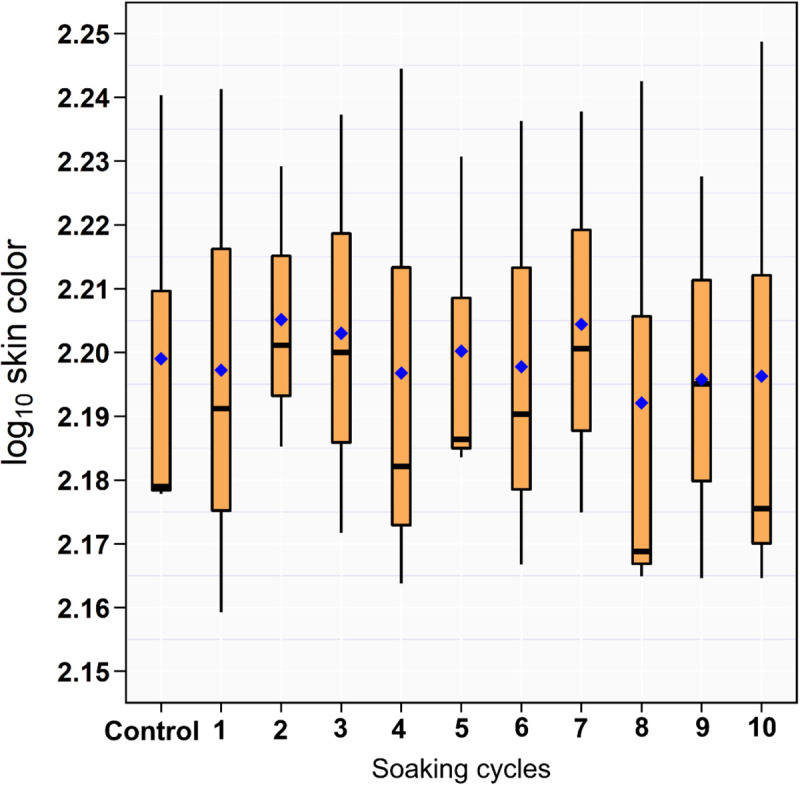
Box and whiskers plot of log_10_ RGB of the drumstick skin color across the 10 sequential soaking cycles.

The results of the RGB measurements showed no difference between the drumsticks treated with ozonated water of 8 ppm and DW (Figure 2).

### Effect of Aqueous O_3_ Volume on *Salmonella* Reduction Using Sequential Spraying Approach

**FIGURE 3:**
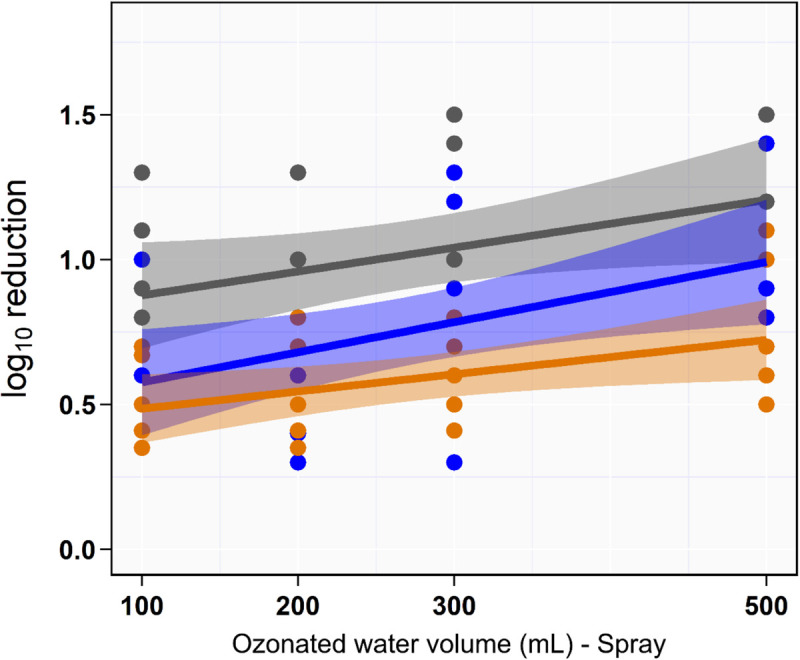
Scatterplot of the linear relationship between volume of aqueous ozone and rate of *Salmonella typhimurium*–*choleraesuis* reduction in spraying water (gray), skin surface (blue), and subcutaneous (orange).

Univariate analysis indicated that the reduction in aSTC cell count when exposed to ozonated water of 8 ppm using spraying approach was not dependent on the volume of ozonated water (*P* = 0.16, *R*^2^ = 0.01; Table 1 and Figure 3). Distinct differences in the aSTC reduction were reported between different sampling locations (spraying water, skin, and SC; *P* ≤ 0.001, *R*^2^ = 0.38; Supplementary Table S2 and Figure 3).

**TABLE 1:** Univariate linear regression model for predicting *Salmonella typhimurium*–*choleraesuis* reduction in spraying water, skin surface, and subcutaneous of drumsticks exposed to 100, 200, 300, and 500 ml ozonated water of 8 ppm.

	**Coefficient**	**Estimated value**	***SE***	**Probability**	**Model *R*^2^**
Spraying	Intercept	0.799	0.086	<0.001	0.101
water	Volume	0.002	0.001	0.129	
Skin	Intercept	0.696	0.091	<0.001	0.001
	Volume	0.001	0.002	0.896	
SC	Intercept	0.409	0.051	0.001	0.140
	Volume	0.002	0.001	0.072	

### *Salmonella* Reduction Using Sequential Spraying Approach

**FIGURE 4:**
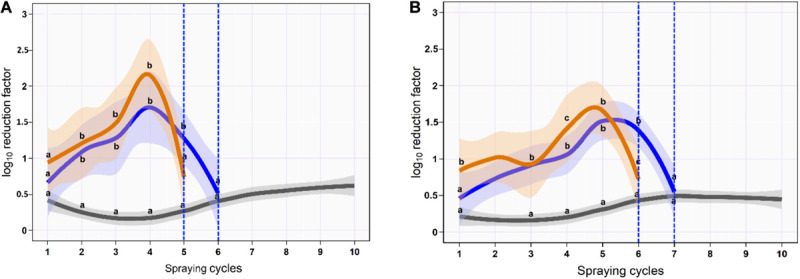
Mean and 95% CI of the log_10_ reduction in *Salmonella typhimurium*–*choleraesuis* (aSTC) cell count on skin surface **(A)** and subcutaneous **(B)** of drumstick sprayed with distilled water (gray), ozone of 8 ppm (blue), and ozone–lactic acid blend (orange) using multi-sequential spraying approach. The vertical dashed blue line indicates that no aSTC was detected.

On the skin surface, the first spraying cycle using ozonated water of 8 ppm reduced the aSTC load from 6.9 to 6.3-log_10_/cm^2^ with an average RF of 0.6-log_10_/cm^2^ (95% CI, 0.4–0.8). The average RF was then gradually increased in the subsequent spraying cycles until it reached a peak of 1.7-log_10_/cm^2^ (95% CI, 0.9–2.5) at the fourth spraying cycle, where the aSTC load reduced from 3.4 to 1.7-log_10_/cm^2^. Six sequential spraying cycles were sufficient to reduce the heavy bioload of aSTC (6.9-log_10_/ml) below the detectable limit (Supplementary Table S3 and Figure 4A). However, O_3_–LA blend reduced the aSTC load from 6.9 to 6.0-log_10_/cm^2^ in the first washing cycle with an average RF of 0.9-log_10_/cm^2^ (95% CI, 0.6–1.3), reaching a peak of 2.2-log_10_/cm^2^ (95% CI, 1.5–2.7) at the fourth spraying cycle, where the aSTC load decreased from 2.9 to 0.7-log_10_/cm^2^. O_3_–LA blend showed higher reduction in the aSTC load than aqueous O_3_ with a total average difference of 0.2-log_10_/cm^2^ (*P* = 0.12), where five sequential spraying cycles were sufficient to reduce the heavy bioload of aSTC below the detectable limit (Supplementary Table S3 and Figure 4A).

In SC, ozonated water of 8 ppm reduced the aSTC load to 0.4-log_10_ (95% CI, 0.1–0.7) in the first spraying cycle, where aSTC load decreased from 6.8 to 6.4-log_10_. The average RF was then gradually increased in the subsequent spraying cycles until it reached a peak of 1.5-log_10_ (95% CI, 1.0–1.9) at the fifth spraying cycle, where the aSTC load was decreased from 3.7-log_1__0_ to 2.2-log_10_. Seven sequential washing cycles were sufficient to reduce the heavy bioload of aSTC (6.8-log_10_/ml) below the detectable limit (Supplementary Table S1 and Figure 1B). However, O_3_–LA blend reduced the aSTC load from 6.8 to 5.9-log_10_ with an average RF of 0.8 (95% CI, 0.4–1.1) in the first spraying cycle, reaching a peak of 1.9-log_10_ (95% CI, 1.4–2.4) at the fifth spraying cycle, where the aSTC load decreased from 2.4-log_1__0_ to 0.7-log_10_. O_3_–LA blend showed higher reduction in the aSTC load than aqueous O_3_ where sex sequential spraying cycles were sufficient to reduce the heavy bioload of aSTC below the detectable limit in SC (Supplementary Table S3 and Figure 4B).

### Effect of *Salmonella* Load on the Reduction Rate

**FIGURE 5:**
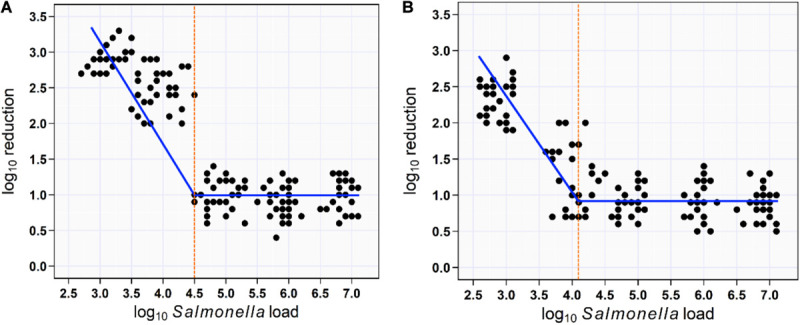
Scatterplot of the relationship between aSTC load and reduction rate. Segmented mixed models regression fitted two lines to the aSTC load–reduction rate relationship and identified a break point (aSTC load = 4.5-log_10_) where the reduction rate increased markedly using multi-sequential soaking approach **(A)**. Segmented mixed models regression fitted two lines to the aSTC load–reduction rate relationship and identified a break point (aSTC load = 4.2-log_10_) where the reduction rate increased markedly using multi-sequential spraying approach **(B)**.

For soaking approach, segmented mixed models regression fitted two lines to the aSTC load–reduction rate relationship for 150 samples (skin surface) and identified a break point at aSTC load = 4.5-log_10_/cm^2^ (95% CI, 1.7–7.3). This break point represented the value for aSTC load where the reduction rate of aSTC increased markedly. The model equations were the reduction rate = 0.96 + 0.04 × aSTC load when aSTC load ≥ 4.5-log_10_/cm^2^ and the reduction rate increased in a linear manner after this cut point, such that: the reduction rate = 0.94 − 0.71 aSTC load. The 95% CI for the slope coefficient (b1) was 22–39 and < 4.5-log_10_/cm^2^. The 95% CI for the second slope was −0.96 to −0.57 (Figure 5A).

For spraying approach, segmented mixed models regression fitted two lines to the aSTC load–reduction rate relationship for 150 samples (skin surface) and identified a break point at aSTC load = 4.2-log_10_ (95% CI, −1.5 to 9.9). This break point represented the value for aSTC load where the reduction rate of aSTC increased markedly. The reduction rate remained constant at 0.8-log_10_ (95% CI, 0.1–2.1) until 4.2-log_10_. The reduction rate increased in a linear manner after this cut point, such that: the reduction rate = 0.8 − 0.9 × aSTC load. The 95% CI for the second slope (−1.1 to −0.7) included 1, indicating that all of the increase in the reduction rate was due to aSTC load (Figure 5B).

## Discussion

In the last few years, a great attention has been provided for the utilization of O_3_ in the poultry processing industry to reduce the contamination with foodborne pathogens, such as *Salmonella* and *Campylobacter* in the final product. The main goal of this study was to evaluate the microbial killing capacity of aqueous O_3_ and O_3_–LA blend on the drumsticks contaminated with heavy bioload of *Salmonella* using multi-sequential soaking or spraying approaches. Additionally, this study aimed to utilize the segmented linear regression to evaluate the effect of microbial load on the microbial killing capacity of O_3_ using soaking or spraying approaches. The first major finding of the study reported here was that the six sequential washing and seven sequential spraying cycles with 8 ppm ozonated water provide an efficient method for reducing 7.0-log_10_ aSTC load-contaminated drumsticks (the worst-case scenario) below the detectable limit. The second major finding was that the addition of 0.3% L-lactate seems to increase the decontamination power of aqueous O_3_ on aSTC-contaminated drumsticks. The third major finding was that the bacterial load on chicken carcass has a significant impact on the microbial killing capacity of aqueous O_3_.

It is important to identify the potential intervention of aqueous O_3_ to meet the bacterial load reduction requirements in the poultry processing industry. Multi-sequential application of antimicrobials is a common approach used in the poultry processing industry to overcome the physical properties of the skin surface that provides a complex surface niche for bacterial colonization, in addition to the opening, exposing channels, and crevices in the chicken skin that protect bacteria from the effects of antimicrobial interventions (Thomas and McMeekin, 1980; Veerkamp, 1990; Franchin et al., 2010; Benli et al., 2011; Tan et al., 2014). To the best of our knowledge, this is the first study that provides information about the microbial killing capacity of ozonated water on the heavily *Salmonella*-contaminated cut up parts using multi-sequential soaking and spraying approaches. The results of this study showed a higher aSTC reduction on the skin surface than SC during the first washing cycle that is considered a sensible result because of the high number of bacteria vulnerable to O_3_ molecules and the physical removal from washing (Franchin et al., 2010; Benli et al., 2011). In the subsequent soaking cycles, the reduction rate was fluctuated. In addition to the physical properties of the skin surface, this is also might be because the organic compounds found on the drumsticks surface prevent the penetration of O_3_ molecules to deeper layers (Restaino et al., 1995), and the high bacterial load makes bacteria accumulate over each other, creating hard penetrating masses (Megahed et al., 2018a,b). The continuous soaking results in decreasing the bacterial load and helping in destroying the complex colloid of bacteria and organic matter and exposing the underneath hidden organisms to O_3_ molecules (Li and Ji, 2017). Six washing cycles of 500 ml of ozonated water of 8 ppm are therefore recommended to decontaminate the drumsticks from high bioload of *Salmonella*. This scenario has been supported by the results of segmented linear regression that showed no association between aSTC load of ≥ 4.5-log_10_ and the reduction rate; however, aSTC load of < 4.5-log_10_ showed significant negative association with the aSTC reduction rate.

Addition of LA seems to increase the microbial killing capacity of aqueous O_3_. To the best of our knowledge, this is the first study to evaluate the decontamination power of low pH ozonated water by adding a low dose of LA on the heavily *Salmonella*-contaminated drumsticks. The antimicrobial power of O_3_ is affected by several factors including O_3_ dose, contact time, temperature, pH, and the presence of organic and inorganic matter (Castillo et al., 2003). Organic acids, such as acetic, lactic, and citric acids, have received the most attention among the sanitizers for their marked bactericidal effects (Dickson and Anderson, 1992). LA seems promising for implementation in the poultry processing facilities because of its availability, cost-effectiveness, ease of use, decontamination potential, and generally recognized as a safe antimicrobial (Ramirez-Hernandez et al., 2018). A decrease in pH of ozonated water by adding LA should intensify the microbial killing capacity of O_3_ through two mechanisms; first, low pH decreases the rate of O_3_ degradation (Gardoni et al., 2012); second, soaking/spraying with low pH ozonated water causes cytoplasmic acidification resulting in malfunction of the energy and regulatory parameters and accumulation of free acid anions that kill or retard the microbial growth and survival (Alonso-Calleja et al., 2004; Cardenas et al., 2011; Sohaib et al., 2016). The average reduction in this study from using O_3_–LA blend is consistent with previous studies that reported an average reduction of 0.8-log_10_/cycle produced by 100 ml ozonated water (8 ppm) in combination with 0.3% LA (pH 2–3) for 15 s exposure time (Dickson and Anderson, 1992; Gardoni et al., 2012; Ramirez-Hernandez et al., 2018). This reduction is considered an efficient reduction level for post-chilling spraying step under commercial condition (Dickson and Anderson, 1992).

The average reductions of 1.5-log_10_/ml on the skin surface and 1.0-log_10_/ml in SC that have been reported in the soaking approach are inconsistent with an earlier study by Sheldon and Brown in 1986 that reported 0.6-log_10_ reduction in *Salmonella* after exposing to ozonated water with a concentration ranging from 3.0 to 4.5 ppm for 25 min exposure (Yang and Chen, 1979). In 2012, Trindade and colleagues reported 2.0–3.0-log_10_ reduction in the *Salmonella*-contaminated chicken carcass after 30 min exposure to 6.0 ppm ozonated water (Trindade et al., 2012). In 1979, Yang and Chen reported a reduction rate of 7.08-log_10_ in *Salmonella* load for ozonated water of 19.0 ppm with a maximum reduction occurring between 2 and 3 min of exposure (United States Department of Agriculture, 2002). It seems that the concentration of O_3_ is a key factor affecting its microbial killing capacity (Megahed et al., 2018a). O_3_ might interact with the organic chemical compounds, such as albumin, that may be present on the chicken carcass surface resulting in consuming the O_3_ molecules and prevent the antimicrobial efficacy from being applied to pathogens. Therefore, decontamination of meat surface requires an additional O_3_ dose rate to have the desired biocide efficacy (Franchin et al., 2010).

The color of chicken carcass is an important quality factor that potentially affects the consumer-purchasing decisions (Sanchez-Escalante et al., 2001). The utilization of O_3_ in the meat industry is challenging because its strong oxidative power can cause damage to meat through the destruction of fatty acids and cellular proteins (Haute et al., 2015). The mechanism of color changes is related to the oxidation of myoglobin with O_3_ molecules producing metmyoglobin that causes discoloration by reducing the redness of meat (Mancini and Hunt, 2005; Bekhit et al., 2013). O_3_ exposure produced changes in the color of beef meat (Stivarius et al., 2002; Cardenas et al., 2011). However, the data of this study did not show any effects of O_3_ on the RGB measurements of drumsticks similar to that observed in several earlier studies (Stivarius et al., 2002; Chen et al., 2014). This might be because the chicken meat is categorized as a white meat due to the low content of myoglobin (Guastalli et al., 2016); therefore, O_3_ has a minimal effect on the skin color appearance of chicken carcass.

The decontamination of chicken carcass in the poultry processing plants mostly occurs at three levels: (1) carcass rinse in the pre-chilling tanks, (2) water chilling step, and (3) spray or drench post-chilling process (Chen et al., 2014). The average exposure to antimicrobials in the poultry processing facilities is approximately 30 min (Guastalli et al., 2016). The average spraying volume of the combination of water and antimicrobials that is mostly used to decontaminate the chicken parts at the post-chilling process is approximately 100–200 ml for 15 s exposure (Ramirez-Hernandez et al., 2018). Interestingly, the data of the spraying protocol reported in this study did not show a significant impact of the ozonated water volume on the aSTC reduction rate. One important concern of food processing industries is water conservation goals (BR50). Therefore, the results of this study suggest reducing the volume of ozonated water in post-chilling spray of drumsticks to meet the USDA requirements for water conservation goals (BR50). The frozen chicken parts showed growth of aerobic bacteria from 2.9 to 3.5-log_10_ CFU/cm^2^ (Yang and Chen, 1979; Jindal et al., 1995). The reduction rate in both studies (Yang and Chen, 1979; Jindal et al., 1995) ranged from 1.0 to 1.2-log_10_ that is consistent with the reduction rate reported in this study. However, both studies used different O_3_ doses, 3.88 ppm of aqueous O_3_ for 20 min (Yang and Chen, 1979) and 0.44–0.54 ppm of aqueous O_3_ for 45 min (Jindal et al., 1995).

In our previous studies, we reported a significant effect of the bacterial load on the microbial killing capacity of ozonated water where we found that there is a direct relationship between the bacterial load and the reduction rate (Megahed et al., 2018a,b). This is a physically sensible relationship as the more bacteria, the more complex bacterial mass formed, the more O_3_ consumed (Megahed et al., 2018a,b). To the best of our knowledge, this is the first study that used the segmented linear regression to determine the level of bacterial load at which the O_3_ molecule has a direct effect on bacteria. This information can serve as a guide to identify the optimal operational conditions of O_3_ that should be used to achieve a safe level of *Salmonella* reduction in the poultry processing facilities. The limitation of this study is that this study is conducted in a laboratory area that is considered a controlled environment. Additionally, this study artificially contaminated the drumsticks end-product with no assessment of the aqueous O_3_ on the naturally *Salmonella*-contaminated chicken, that is considered a different story because of the attachment of *Salmonella* to the skin surface and inside the feather follicles. Therefore, additional studies are indicated to determine the external validity of the results.

## Conclusion

Six sequential soaking and seven sequential spraying cycles with ozonated water of 8 ppm provide an efficient safe protocol to reduce the high *Salmonella* load that contaminated the skin surface and SC of chicken parts below the detectable limits. Addition of LA to ozonated water seems to enhance the decontamination power of O_3_. Bacterial load is an important factor controlling the microbial killing capacity of O_3_ on the surface of chicken carcasses.

## Data Availability Statement

The original contributions presented in the study are included in the article/Supplementary Material, further inquiries can be directed to the corresponding author/s.

## Author Contributions

AM did conceptualization, data curation, formal analysis, investigation, methodology, software, validation, visualization, and writing original draft. BA did supervision, methodology, visualization, and review & editing. JL did conceptualization, data curation, formal analysis, investigation, methodology, project administration, resources, software, supervision, validation, visualization, and writing original draft. All authors contributed to the article and approved the submitted version.

## Conflict of Interest

The authors declare that the research was conducted in the absence of any commercial or financial relationships that could be construed as a potential conflict of interest.
