# Supplementary material for: Antimicrobial Efficacy of Aqueous Ozone and Ozone–Lactic Acid Blend on Salmonella-Contaminated Chicken Drumsticks Using Multiple Sequential Soaking and Spraying Approaches
Source: Front Microbiol. 2020 Dec 14;11:593911. doi: 10.3389/fmicb.2020.593911 (PMC7768038; doi:10.3389/fmicb.2020.593911)
Supplement: Supplementary file 1 [file Data_Sheet_1.docx]

**SupplementaryTable 1.**Median and range for cell forming unit (cfu) of *Salmonella*in the soaking water, and on skin surface and subcutaneousof control, ozone (O_3_), and ozone-lactic acid blend (O_3_-LA) treated drumsticks.

| **Washing cycles** | **Washing water(log_10_/mL)** | | | **Skin surface(log_10_/cm^2^)** | | | **Subcutaneous (log_10_/cm^2^)** | | |
| --- | --- | --- | --- | --- | --- | --- | --- | --- | --- |
|  | **Control** | **O_3_** | **O_3_-LA** | **Control** | **O_3_** | **O_3_-LA** | **Control** | **O_3_** | **O_3_-LA** |
| **1^st^wash** | 7.0 (6.7,7.0)^Aa^ | 5.2 (4.5,6.5)^Ba^ | 4.8 (4.2,6.1)^Ba^ | 6.4 (5.5,6.9)^Aa^ | 5.7 (4.7,6.2)^Ba^ | 5.2 (4.1,6.3)^Ca^ | 6.7 (6.1,6.9)^Aa^ | 6.1 (4.8,6.8)^Ba^ | 5.8 (5.1,6.8)^Ba^ |
| **2^nd^wash** | 6.8 (5.8,7.1) ^Aa^ | 2.9  (2.2,6.0)^Bb^ | 2.8  (2.0,4.5)^Bb^ | 5.9 (5.1,6.7)^Ab^ | 3.9 (3.4,5.1)^Bb^ | 3.8 (3.0,4.7)^Bb^ | 6.4 (5.8,6.9)^Aa^ | 5.1 (3.5,5.6)^Bb^ | 4.6 (3.5,5.6)^Bb^ |
| **3^th^wash** | 6.3 (5.0,6.9)^Aa^ | 2.1 (0.0,3.4)^Bc^ | 0.0 (0.0,2.4)^Cc^ | 5.5 (5.0,6.5)^Ab^ | 2.9 (2.0,4.1)^Bc^ | 2.0  (0.0,3.0)^Cc^ | 6.2 (5.5,6.6)^Aa^ | 3.8 (3.0,4.9)^Bc^ | 3.4  (2.0,4.1)^Bc^ |
| **4^th^wash** | 6.2 (4.5,6.9)^Aa^ | 0.0  (0.0,2.1)^Bd^ | 0.0^Bc^ | 5.3 (4.5,5.9)^Ab^ | 0.0 (0.0,2.0)^Bd^ | 0.0 ^Bd^ | 5.7 (5.1,6.1)^Ab^ | 2.5 (2.1,3.0)^Bd^ | 2.0  (0.0,2.7)^Cd^ |
| **5^th^ wash** | 5.5 (6.0,6.8)^a^ | 0.0 | 0.0 | 5.0  (4.2,5.8)^Ab^ | 0.0 ^Bd^ | 0.0 | 5.4  (5.0,5.7)^Ab^ | 2.0 (0.0,2.4)^Bd^ | 0.0 ^Be^ |
| **6^th^ wash** | 5.3  (4.0,6.6)^a^ | 0.0 | 0.0 | 4.5  (3.5,4.8)^Ac^ | 0.0 | 0.0 | 4.8  (3.9,5.7)^Ac^ | 0.0 ^Be^ | 0.0 ^Bf^ |
| **7^th^ wash** | 5.0  (3.9,6.0)^a^ | 0.0 | 0.0 | 3.7  (3.0,4.2)^Ad^ | 0.0 | 0.0 | 4.5  (3.5,5.5)^Ac^ | 0.0 | 0.0 |
| **8^th^ wash** | 4.2  (3.2,5.5)^b^ | 0.0 | 0.0 | 3.0  (2.1,4.0)^Ae^ | 0.0 | 0.0 | 4.3  (3.3,5.3)^Ac^ | 0.0 | 0.0 |
| **9^th^ wash** | 3.5  (3.0,5.0)^b^ | 0.0 | 0.0 | 2.3  (2.0,3.7)^Af^ | 0.0 | 0.0 | 4.0  (2.7,4.9)^Ad^ | 0.0 | 0.0 |
| **10^th^ wash** | 3.1  (2.3,4.7)^c^ | 0.0 | 0.0 | 2.0  (0.0,3.2)^Ag^ | 0.0 | 0.0 | 3.0  (2.5,4.5)^Ae^ | 0.0 | 0.0 |

^A-C^Values within a row are significantly different between control and treated groups for each type of sampling method (*P*<0.05).

^a-g^Values with different letters within a column are significantly different of two sequential cycles (*P*<0.05).

**Supplementary Table 2.**Median and range of cell forming unit (cfu) of *Salmonella* Typhimurium-Choleraesuis in washing water, skin surface, and subcutaneous of control and ozone treated drumsticks. Drumsticks were sprayed with 100, 200, 300, and 500 mL ozonated water of 8 ppm for 4 minutes exposure.

|  | **Washing water(log_10_/mL)** | | **Skin surface(log_10_/cm^2^)** | | **Subcutaneous (log_10_/cm^2^)** | |
| --- | --- | --- | --- | --- | --- | --- |
|  | **Control** | **Ozone** | **Control** | **Ozone** | **Control** | **Ozone** |
| Spray-100 mL | 7.0 (7.0,7.1)^a^ | 6.2 (6.0,6.8)^*b^ | 6.9  (6.7,7.0)^a^ | 6.5  (5.8,6.9)^*a^ | 6.9  (6.9,7.0)^a^ | 6.6  (6.4,6.7)^*a^ |
| Spray-200 mL | 6.9 (6.7,7.0)^a^ | 6.3 (6.1,6.5)^*a^ | 7.0 (6.7,7.1)^a^ | 6.0 (5.2,6.2)^*a^ | 7.0 (6.9,7.0)^a^ | 6.4 (6.1,6.8)^*a^ |
| Spray-300 mL | 6.8 (6.6,6.9)^a^ | 5.9 (5.5,6.1)^*a^ | 6.8 (6.6,6.9)^a^ | 5.7 (5.0,5.9)^*a^ | 6.8  (6.8,7.0)^a^ | 6.5  (6.0,6.7)^*a^ |
| Spray-500 mL | 6.6 (6.3,6.7)^a^ | 4.8  (4.4,5.2)^*a^ | 6.8 (6.6,6.9)^a^ | 5.3 (4.2,5.9)^*a^ | 7.0  (6.9,7.0)^a^ | 6.3  (5.9,6.5)^*a^ |

*Values within a row are significantly different between control and treated groups for each type of sampling (*P*<0.001).

^a-c^Values with different letters within a column are significantly different (*P*<0.05).

**Supplementary Table 3.**Median and range for cell forming unit (cfu) of control and ozone treated drumsticks in washing water, skin surface, and subcutaneous. Drumsticks was sprayed with 50 mL ozonated water of 8 ppm for 4 minutes exposure.

| **Spraying cycles** | **Washing water(log_10_/mL)** | | | **Skin surface(log_10_/cm^2^)** | | | **Subcutaneous (log_10_/cm^2^)** | | |
| --- | --- | --- | --- | --- | --- | --- | --- | --- | --- |
|  | **Control** | **O_3_** | **O_3_-LA** | **Control** | **O_3_** | **O_3_-LA** | **Control** | **O_3_** | **O_3_-LA** |
| **1^st^wash** | 7.0 (6.8,7.0)^Aa^ | 6.1 (5.5,6.8)^Ba^ | 5.9 (4.8,6.5)^Ba^ | 6.7 (6.1,6.9)^Aa^ | 5.9 (5.5,6.5)^Ba^ | 5.6 (5.1,6.2)^Ba^ | 6.8 (6.4,7.0)^Aa^ | 6.4 (5.1,6.8)^Ba^ | 6.1 (5.2,6.8)^Ba^ |
| **2^nd^wash** | 6.9  (6.7,7.0) ^Aa^ | 5.1  (4.0,5.7)^Bb^ | 4.5  (3.5,5.4)^Cb^ | 6.4 (5.7,6.9)^Aa^ | 4.6 (4.0,5.5)^Bb^ | 4.4 (3.2,5.7)^Bb^ | 6.7 (6.1,7.0)^Aa^ | 5.5 (4.8,6.7)^Bb^ | 4.7 (4.1,5.7)^Cb^ |
| **3^th^wash** | 6.7 (6.5,7.0)^Aa^ | 3.7 (2.4,5.5)^Bc^ | 2.7 (0.0,3.7)^Cc^ | 6.2 (5.5,6.9)^Aa^ | 3.5 (2.5,4.2)^Bc^ | 2.8  (2.1,3.8)^Cc^ | 6.4 (5.9,6.9)^Aa^ | 4.5 (3.5,6.1)^Bc^ | 4.1  (2.5,5.2)^Cc^ |
| **4^th^wash** | 6.5 (6.3,6.8)^Aa^ | 2.5  (0.0,3.5)^Bd^ | 0.0  (0.0,2.5)^Cd^ | 6.0 (5.4,6.9)^Aa^ | 2.1 (0.0,3.0)^Bd^ | 0.0 (0.0,2.5)^Cd^ | 6.2 (5.4,6.8)^Aa^ | 3.8 (2.5,4.8)^Bd^ | 2.5  (2.1,3.4)^Cd^ |
| **5^th^ wash** | 6.4 (6.0,6.7)^Aa^ | 0.0  (0.0,2.4)^Be^ | 0.0  (0.0,2.1)^Bd^ | 5.8  (5.3,6.2)^Aa^ | 0.0 (0.0,2.1)^Be^ | 0.0^Be^ | 5.9  (5.4,6.8)^Aa^ | 2.0 (2.0,3.0)^Be^ | 0.0  (0.0,2.0)^Ce^ |
| **6^th^wash** | 6.1  (5.7,6.5)^Aa^ | 0.0^Bf^ | 0.0^Bd^ | 5.2  (4.5,6.8)^Ab^ | 0.0^Be^ | 0.0^Be^ | 5.6  (4.7,6.2)^Ab^ | 0.0  (0.0,2.0)^Bf^ | 0.0^Cf^ |
| **7^th^wash** | 5.7  (5.2,6.0)^a^ | 0.0 | 0.0 | 5.0  (3.5,6.0)^Ac^ | 0.0 | 0.0 | 4.9  (4.2,5.9)^Ac^ | 0.0 ^Bf^ | 0.0 |
| **8^th^wash** | 5.1  (4.1,5.7)^b^ | 0.0 | 0.0 | 4.1  (3.2,5.5)^Ad^ | 0.0 | 0.0 | 4.5  (4.0,5.2)^Ad^ | 0.0 | 0.0 |
| **9^th^wash** | 4.5  (3.6,4.9)^c^ | 0.0 | 0.0 | 3.7  (3.1,5.0)^Ae^ | 0.0 | 0.0 | 4.0  (3.5,4.9)^Ae^ | 0.0 | 0.0 |
| **10^th^wash** | 4.0  (2.9,4.2)^d^ | 0.0 | 0.0 | 3.0  (2.5,4.1)^Af^ | 0.0 | 0.0 | 3.5  (3.0,4.6)^Af^ | 0.0 | 0.0 |

^A-C^Values within a row are significantly different between control and treated groups for each type of sampling method (*P*<0.05).

^a-g^Values with different letters within a column are significantly different of two sequential cycles (*P*<0.05).
